# Past-year gambling behaviour among patients receiving opioid substitution treatment

**DOI:** 10.1186/1747-597X-10-4

**Published:** 2015-01-27

**Authors:** Sari Castrén, Anne H Salonen, Hannu Alho, Tuuli Lahti, Kaarlo Simojoki

**Affiliations:** Department of Tobacco, Gambling and Addiction, National Institute for Health and Welfare, P.O. Box 30, FIN-00271 Helsinki, Finland; Internal Medicine, University of Helsinki, Helsinki, Finland; Faculty of Social Sciences, Department of Behavioural Sciences and Philosophy, University of Turku, Turku, Finland; A-clinic Foundation, Maistraatinportti 2, 00240 Helsinki, Finland

**Keywords:** Gambling, Opioid substitution treatment, Buprenorphine-naloxone, Methadone, Substance abuse

## Abstract

**Background:**

Substance abuse and gambling problems are associated, however, studies on gambling problems among opioid substitution treatment (OST) patients are scarce. The aims of this study are to explore the association of gender, age, treatment medication and treatment program with gambling behaviour, including gambling participation and gambling problems, among OST patients.

**Findings:**

All OST patients (n = 244) in three Finnish outpatient clinics were recruited in March - April 2014. The response rate was 64.3%. OST programs included two choices of orientation (rehabilitative/harm reduction) and two choices for treatment medication (methadone/buprenorphine-naloxone). Of 144 respondents, 70.1% had gambled during the past year and 12.5% were identified as potential past-year problem gamblers. Gambling was statistically significant more commonly among males (79.8%) compared with females (53.7%). Similarly patients in the rehabilitative program gambled (75.9%) more than those in the harm reduction program (50.0%). Gender, age, treatment medication or treatment program was not associated with past-year gambling problems.

**Conclusions:**

Gambling participation of the OST patients seemed to be somewhat similar compared with the Finnish general population, but gambling problems were more common among OST patients. Gender and age may not be very strong indicators of risk while screening problem gamblers among OST patients. Institution of a problem gambling screening program is recommended, and additional intervention for gambling problems should be implemented for that need as a part of OST.

## Introduction

Opioid addiction, as in other addictions, is characterized by behaviours including one or more of the following manifestations related to drug use: impaired control, compulsive use, craving and continued use regardless of harm [1]. These diagnostic manifestations resemble those of gambling disorder (GD) as categorized by the DSM-5 [1]. Both substance use disorder (SUD) and GD present a loss of control over a person’s behaviour and also have negative personal, vocational and social consequences.

The prevalence of opioid abuse worldwide is 0.4% [2] while the standardized problem gambling prevalence rate varies from 0.5% to 7.6% [3]. Among misusers, however, the prevalence of GD is considerably higher varying from 8% to 21% and even higher (17% to 27%) among patients in methadone maintenance treatment (MMT) [4, 5]. Based on both population studies and studies from the clinical context, males and people younger than 35 years gamble more and have more gambling problems than females or people 35 years or older [6–8]. Characteristically, patients with opioid dependence also have other comorbidities: typically either problems with other substances or co-occurring psychiatric disorders such as depression or anxiety and personality disorders [9–11]. Similarly, patients with GD seem to have a high rate of co-occurring SUD and psychiatric disorders [4, 12, 13].

Based on previous studies, both GD and SUD may be present as a primary addiction: GD may precede SUD or vice versa [14, 15]. However, only a few studies have addressed the issue of a concomitant GD within SUD patients such as patients within opioid substitution treatment (OST) [5, 15, 16].

In Finland, the rehabilitation oriented OST focuses on abstinence and psychosocial rehabilitation using structured treatment programs. The harm reduction approach focuses on the patient’s quality of life and consists of services promoting safer use of drugs and injection facilities, overdose prevention, social and health issues, and peer support. The harm reduction approach is used with patients who are currently not able to quit drug use. The patients are selected to the different programs according to the severity of addiction, psychiatric comorbidities and assumed capability of rehabilitation. Methadone is the choice for more severe patients, who have to attend the clinic daily. In both of the orientations, the choice of treatment medication (methadone/buprenorphine-naloxone) is based on an individual assessment and tailored to the needs of each patient [17].

In order to better treat the patients with dual diagnosis of both opioid addiction and GD, more research is needed to understand how these disorders are related and how the concomitant presence of these disorders possibly affect their treatment. The aims of this study are to explore the association of gender, age, treatment medication and treatment program with gambling behaviour, including gambling participation and gambling problems, among OST patients.

## Methods

Cross–sectional data (n = 144) was based on a total sample of 224 OST patients treated between March and April 2014 at three outpatient clinics in Finland. The response rate of the study was 64.3%. The data regarding gambling behaviour were collected as a part of clinical work and used in this study with the approval of the Ethical Committee of the A-Clinic Foundation.

Past-year gambling participation was inquired using a question: “Have you gambled during the past 12 months?” with yes/no answers. Past-year gambling problems were assessed using the Brief Biosocial Gambling Screen (BBGS) [18], a three-item scale measuring neuro-adaptation, psychosocial characteristics and adverse social consequences of gambling (Table 1) with Cronbach Alpha of 0.70. In the instructions gambling was defined as follows: “Gambling means games you can play with money, for example, lotteries, Keno, slot machine games, internet gambling (e.g. internet poker) and horse trotting games.”

**Table 1:** **The proportion of endorsed criteria for problem gambling among the patients (n = 144) treated at the outpatient clinics**

Criteria	Question	n (%)
**1. Neuro-adaptation:**	“During the past 12 months, have you become restless, irritable or anxious when trying to stop/cut down on gambling?”	13 (9.0)
**2. Psychosocial characteristics**	“During the past 12 months, have you tried to keep your family or friends from knowing how much you gambled?”	7 (4.9)
**3. Adverse social consequences of gambling**	“During the past 12 months did you have such financial trouble as a result of your gambling that you had to get help with living expenses from family, friends or welfare?”	8 (5.6)
Past-year gambling problems*		18 (12.5)

BBGS, Brief Biosocial Gambling Screen; The response options included yes and no. *One or more positive answers (yes) to the questions indicated potential past-year gambling problems.

Independent variables included gender, age, orientation of the treatment program (rehabilitative/harm reduction) and the type of treatment medication (methadone/buprenorphine-naloxone).

Two formulations of sublingual tablets of buprenorphine: mono-buprenorphine (Subutex^®^), supplied as 0.4 mg, 2 mg and 8 mg tablets and buprenorphine-naloxone (Suboxone^®^), supplied as 2 mg (buprenorphine)/0.5 mg (naloxone) and 8 mg (buprenorphine)/2 mg (naloxone). Administration of the medication in the harm reduction group was supervised 5–7 times per week and in the rehabilitative group at least once a week, depending on the patient’s individual treatment plan. Treatment retention among OST patients in Finland is up to 80% after 18-month follow-up [19, 20].

The data were analysed using SPSS 21.0 software (SPSS, Inc., Chicago, IL, USA). Statistical significance (p) was determined using the Fisher’s exact test. The Odds Ratio (OR) was calculated separately for each variable.

## Findings

A total of 144 patients (62.2% males) participated in this study (Table 2). The mean age for males was 36.6 (SD 7.0, range 22–55) and for females 34.7 (SD 9.0, range 22–59). The patients in the rehabilitative program were younger than the patients in the harm reduction program.

**Table 2:** **Description of the respondents (n = 144) by the treatment program**

	All n (%)	Rehabilitative n (%)	Harm reduction n (%)
**Gender**			
Males	89 (62.2)	72 (80.9)	17 (19.1)
Females	54 (37.8)	40 (74.1)	14 (25.9)
**Age**			
≤24 years	6 (4.2)	6 (100.0)	-
25-34 years	64 (44.4)	56 (87.5)	8 (12.5)
35-44 years	54 (37.5)	39 (72.2)	15 (27.8)
≥45 years	20 (13.9)	11 (55.0)	9 (45.0)
**Treatment medication**			
Methadone	71 (49.3)	47 (66.2)	24 (33.8)
Buprenorphine-naloxone	73 (50.7)	65 (89.0)	8 (11.0)

77.8% participated in the rehabilitative treatment program while 22.2% participated in the harm reduction program. 49.3% used methadone and 50.7% used buprenorphine-naloxone as the treatment medication (Table 2).

70.1% had gambled during the past year. Past-year gambling was more common among males (79.8%) compared with females (53.7%, p < 0.001, OR 3.40) (Table 3). Past-year gambling was more common among patients in the rehabilitative program (75.9%) compared with patients in the harm reduction program (50.0%, p = 0.008). The OR of being a gambler in the rehabilitative program was 3.15. There was no statistically significant difference in past-year gambling between the two age groups.

**Table 3:** **Association between the correlates and past-year gambling among the patients**

	Gambling n (%)	No gambling n (%)	Significance	Odds ratio
**Gender**			p < 0.001	
Males	71 (79.8)	18 (20.2)		3.40
Females	29 (53.7)	25 (46.3)		a
**Age**			p = 0.368	
<35 years	52 (74.3)	18 (25.7)		1.47
≥35 years	49 (66.2)	25 (33.8)		a
**Treatment medication**			p = 0.203	
Methadone	46 (64.8)	25 (35.2)		1.66
Buprenorphine-naloxone	55 (75.3)	18 (24.7)		a
**Treatment program**			p = 0.008	
Rehabilitative	85 (75.9)	27 (24.1)		3.15
Harm reduction	16 (50.0)	16 (50.0)		a
**Treatment combination**			p = 0.024	
Rehabilitative with methadone	36 (76.6)	11 (23.4)		3.27
Rehabilitative with buprenorphine-naloxone	49 (75.4)	16 (24.6)		3.06
Harm reduction with either medication	16 (50.0)	16 (50.0)		a

Significance is determined by Fischer’s exact test; Odds Ratio is calculated separately for each variable; a, reference group.

Based on the BBGS, 12.5% of the respondents had scores indicating potential gambling problems (Table 1). The criteria measuring neuro-adaptation was endorsed most commonly (9.0%), while 4.9% endorsed the criteria measuring psychosocial characteristics and 5.6% endorsed adverse social consequences of gambling.

There were more males with gambling problems (14.8%) than females (11.2%) (Table 4). There were also more 35 years or older patients (13.5%) with gambling problems than younger than 35 years (11.4%). Respondents in the rehabilitative program (14.3%) had more gambling problems than those in the harm reduction program (6.2%). Gambling problems were more common among those using methadone (15.5%) than those using buprenorphine-naloxone (9.6%). 21.3% of the respondents in the rehabilitative program using methadone had gambling problems (OR 4.05), which was higher than in other treatment combinations. None of these differences were statistically significant.

**Table 4:** **Association between the correlates and past-year gambling problems among patients treated at the outpatient clinics**

	Gambling problem* n (%)	No gambling problems n (%)	Significance	Odds ratio
**Gender**			p = 0.606	
Males	8 (14.8)	79 (85.2)		1.37
Females	10 (11.2)	46 (88.8)		a
**Age**			p = 0.803	
<35 years	8 (11.4)	62 (88.6)		a
≥35 years	10 (13.5)	64 (86.5)		1.21
**Treatment medication**			p = 0.322	
Methadone	11 (15.5)	60 (84.5)		1.73
Buprenorphine-naloxone	7 (9.6)	66 (90.4)		a
**Treatment program**			p = 0.363	
Rehabilitative	16 (14.3)	96 (85.7)		2.50
Harm reduction	2 (6.2)	30 (93.8)		a
**Treatment combination**			p = 0.112	
Rehabilitative with methadone	10 (21.3)	37 (78.7)		4.05
Rehabilitative with buprenorphine-naloxone	6 (9.2)	59 (90.8)		1.53
Harm reduction with either medication	2 (6.2)	30 (93.8)		a

The data (n = 144); *BBGS = 1+, Brief Biosocial Gambling Screen: one or more positive answers (yes) indicated potential past-year gambling problems; Significance is determined by Fischer’s exact test; Odds Ratio is calculated separately for each variable; a, reference group.

## Discussion

The proportion of past-year gamblers among OST patients was at a slightly lower level than in the Finnish general population sample. However, the past-year prevalence of gambling problems among OST patients was clearly higher (12.5%) than at the population level (2.7%) [21], being in line with previous studies [5, 14, 16]. Yet, it is important to notice that previous studies assessed severity of gambling using a different instrument and time frame to this study. Weinstock and colleagues [16] used a self-report survey with the South Oaks Gambling Screen (SOGS) of both lifetime and past two months time frame. Peles and colleagues [5] used SOGS as a lifetime measure, whereas Ledgerwood and Downey [16] altered SOGS to assess the past three months severity of gambling. Time frame, in particular, is an important factor while comparing different studies, since the lifetime measure of gambling is not sensitive to current gambling problems due to false positive answers [22], whereas the past two and three months are specifically addressing the current situation. We used BBGS, which has good psychometric properties [18]. An instrument with a 12-month time frame was selected to exclude the participants in sustained remission of GD [1]. Adding another measure alongside the BBGS would be worth considering in the future.

Neuro-adaptation was the most endorsed criterion in the BBGS. This criterion refers to the behavioural manifestations of withdrawal – irritability and anxiety upon cessation of gambling. This can reflect the known similarities of SUD and GD [23, 24]. The endorsement of this particular criterion may also reflect the characteristics of this particular population, where symptoms of withdrawal may overlap another addiction.

Our results confirmed that male patients in OST gamble significantly more than female patients [5]. However, this particular gender difference was not statistically significant. Therefore, among OST patients, gender may be less associated with GD than at the population level and is not necessarily a moderator of gambling behaviour as compared to, for example, chemical addictions [7].

Patients in OST did not differ from the general Finnish population regarding participation in gambling activities [21]. Patients who were younger than 35 years gambled more often than the 35 years or older patients. However, the proportion of 35 years or older patients categorized as probable problem gamblers was higher than that of the younger ones. Even though these differences did not attain statistical significance this may reflect the characteristics of the patients in OST in general, them being somewhat over 30 years old [5], which in turn differ with the findings among the general population [21].

The patients in the rehabilitation oriented program gambled significantly more and had more gambling problems than the patients in the harm reduction program. Could the patients in the rehabilitative program, with the goal of eventually quitting all drug use, and consequently having less euphoric experience due to treatment, be chasing the euphoria by gambling? SUD and GD co-occur commonly and may manifest either on a behavioural level (for example, behavioural addiction may be fuelled by substance use) or on a syndromal level (for example, a behavioural addiction starts after SUD treatment) [24].

Understanding the similarities, as well as the interaction between GD and SUD, is important in developing treatments especially for these two addictions occurring simultaneously. It can be hypothesised that the patients attending the rehabilitative program may have an increased risk to develop a behavioural addiction (i.e. GD), as a replacement of the opioid addiction. As some studies [25–28] suggest, there may be a behavioural interaction between GD and SUD. On the other hand, it may be explained by the theory of a common liability to addiction [29]. In both GD and SUD reduced control over strong behaviour and urge for immediate rewards [30] have been identified. Conversely, the patients in the harm reduction program may have limited interest in gambling activities, as well as capabilities (e.g. economic, social and cognitive) in other activities due to the severity of their primary diagnosis.

Buprenorphine-naloxone was used more often than methadone, especially among the patients attending the rehabilitative program. Patients in a rehabilitative program with buprenorphine-naloxone had less gambling problems compared to those in a rehabilitative program with methadone. In turn, the patients receiving methadone seem to have more gambling problems. Thus, could buprenorphine-naloxone formulation Suboxone^®^ have an effect in decreasing gambling urges [31, 32], since it contains 10% of naloxone [33]?

This study is limited by the small sample size, the limited number of background variables (e.g. socio-demographic characteristics, type of gambling, comorbid conditions) and the bivariate analysis. In addition, parallel use of other psychoactive drugs, which may be associated with problematic gambling was not recorded here, thus future studies should address this issue. However, the topic is largely unexplored. The data are unique and represent well the patients treated in the outpatient clinics. The number of patients, including the amount of females, who attended the treatment, represents around 5% of the total number of OST patients in Finland. Their background is also representative, thus not reported here. The clinics represent two different major treatment programs used in Finland [19, 20]. However, some of our results did not reach statistical significance, thus a power analysis is recommended in future studies. Gambling problems were measured using a translated and back-translated Finnish version of the BBGS [18], which has not yet been validated in the Finnish or OST context. This study mainly offers valuable suggestive information to clinical practice, includes two relevant lines of treatment approaches (those based on opioid withdrawal and those based on agonist maintenance) [34] and recommendations for further research.

## Conclusions

Gambling participation of the patients in OST seems to be somewhat similar compared with the Finnish general population, but gambling problems were more common among the patients in OST. The gender and age may not be very strong indicators of risk among patients in OST. Assessment of possible gambling problems should be included in clinical practice, and additional intervention in gambling problems should be implemented for those in need as a part of OST. A comprehensive system of screening, brief intervention and treatment referral as a part of a public health approach, for example similar to SBIRT (http://www.samhsa.gov/sbirt), should also be implemented for behavioural addictions occurring alone or with substance use disorders. It has already been noted [14] that OST patients who have concomitant gambling problems may benefit from additional support.
